# Multidisciplinary residential home intervention to improve outcomes for frail residents

**DOI:** 10.1186/s12913-021-07407-y

**Published:** 2022-01-12

**Authors:** Anna Steel, Helen Hopwood, Elizabeth Goodwin, Elizabeth L. Sampson

**Affiliations:** 1 Barnet Hospital, Wellhouse Lane, Barnet, EN5 3DJ UK; 2 Barnet Enfield and Haringey Mental Health Trust, North Middlesex University Hospital, Sterling Way, London, N18 1QX UK; 3 Brunswick Park Medical Centre, Brunswick Park Road, London, N11 1EY UK; 4 Marie Curie Palliative Care Research Department, Division of Psychiatry, UCL, 6th Floor. Wing B. Maple House, 149 Tottenham Court Road, London, W1T 7NF UK

**Keywords:** Interdisciplinary, Long-term care, Geriatric assessment, Mental-health

## Abstract

**Background:**

Residential homes provide accommodation and assistance with personal care only and are not required to have registered nurses on site. However, their residents often have a combination of comorbidity, polypharmacy, frailty and mental-health conditions with poor access to healthcare to meet these needs. Integrated healthcare for older people is a key NHS priority in the Long-Term Plan and the Five-Year Forward View. We describe development and implementation of multi-disciplinary intervention to integrate healthcare and promote interprofessional education.

**Methods:**

A multi-disciplinary residential home quality improvement project in two cycles by a team comprising senior and trainee general practitioners, trainees in geriatrics, psychiatry, pharmacist and residential home senior staff. The intervention was underpinned by the framework for enhanced health in care homes including Comprehensive Geriatric Assessment (CGA) and mental-health review. Each intervention session included an educational presentation by a team member consideration of each resident in a pre-evaluation multi-disciplinary discussion followed by a structured clinical assessment and discussion of proposed management.

**Results:**

Three residential homes participated with a total 34 residents receiving intervention. In one residential home, there was a 75% reduction in admissions for those reviewed and a reduction in overall admission costs. Polypharmacy was reduced by an average of 2 medications per resident across the three sites. There was a 63% increase in cardio-pulmonary resuscitation decisions and 76% increase in advance care planning discussions.

**Conclusion:**

This was an effective model for multi-disciplinary trainees working with a perceived impact on physical and mental health, and valuable opportunities for sharing learning.

## Background

Approximately 420,000 older people live in care homes in England [1]. “Care homes” is a generic term; within the UK there are “care homes with nursing” and “residential homes” (RHs) providing accommodation and support with activities of daily living, but without on-site nurses. Resident needs in both nursing and RHs are complex, with a combination of physical frailty and mental health conditions [2].

Care home residents have 40–50% more emergency admissions and fewer outpatient appointments than the general population of the same age [3]. Many avoidable admissions are driven by unsatisfactory provision of healthcare services [4, 5] with medication errors occurring in approximately 70% of residents [6]. The majority of care home residents are in their last years of life [3] but may not receive adequate end-of-life care or advance care planning (ACP) [7]. This is especially challenging for the 80% of the care home population with dementia [8].

Enhanced Health in Care Homes (EHCH) is a cornerstone of the NHS Long Term Plan [9]. Integrated care between primary, secondary and community providers, is effective and can lower rates of emergency bed use [3, 10]. Multi-disciplinary teams (MDTs) linking community and hospital-based services increase functioning, reduce hospital costs and lengths of stay [11]. Key Recommendations from The British Geriatrics Society (BGS) Quest for Quality report include ensuring fair access to NHS services, providing a comprehensive geriatric assessment (CGA), promoting autonomy and care plans towards the end-of-life [5]. Despite many “top down” policy drivers, implementing change in practice is challenging and a more “bottom-up” context-specific approach such as local quality improvement may deliver more pragmatic and sustainable change [11]. The strength of a quality improvement approach includes linking useful interventions with improvement results and why they worked, which may be able to inform improvement elsewhere [12].. The process of healthcare improvement is about bridging gaps within the six domains of healthcare quality as set out by the Institute of Medicine: safety, effectiveness, patient-centred, timely, efficiency and equity [13].

RHs have higher ambulance call and emergency admission rates [3, 14] and fewer General Practitioner (GP) visits [15] compared to care homes with nursing thus our focus on RHs for this project.

We aimed to address a range of important clinical issues to improve multi-disciplinary care in RHs focusing on key elements derived from current policy; CGA, medication optimisation, end-of-life care planning and education to reduce hospital admissions [16].

## Methods

We conducted a multi-disciplinary RH quality improvement project in two cycles. The first cycle was a pilot, subsequently rolled out to another location. Cycle one was conducted between 20/09/2017–14/02/2018 and cycle two between 21/03/2018–12/08/2018.

### Context

The project was set within three urban RHs in North London. The first cycle was conducted in RHs 1 and 2 and the second cycle in RH 3. The general practice supporting homes 1 and 2 had five GP partners and two salaried GPs looking after 10,500 patients, 125 of whom were in three different RHs. The GP supporting RH 3 worked in a practice with two partners, four salaried GPs and 9000 patients, 40 of whom resided in one RH. We collected data on RH characteristics; location, type of care, number of beds, Care Quality Commission (CQC) rating,

### Intervention team

Cycle one: The team included a GP lead, senior trainees from GP (one), psychiatry (one) and geriatrics (two), a primary-care pharmacist, the RH manager (homes 1 and 2) and deputy manager (home 1).

Cycle two: The team included a GP partner, senior trainees from GP (three), psychiatry (one) and geriatrics (three), primary-care pharmacists and the RH manager.

### Intervention

The project was underpinned by the EHCH implementation framework [16] includes seven key standards; enhanced primary care support, multi-disciplinary support, high quality end-of-life care and dementia care, joined-up commissioning and collaboration between health and social care and workforce development. The EHCH model aims to implement these care elements in a coordinated sustainable way to deliver person centred care. These standards informed the session design which was further adapted for cycle 2 based on the change in setting and learning from cycle 1.

### Cycle 1

**Fig. 1:**
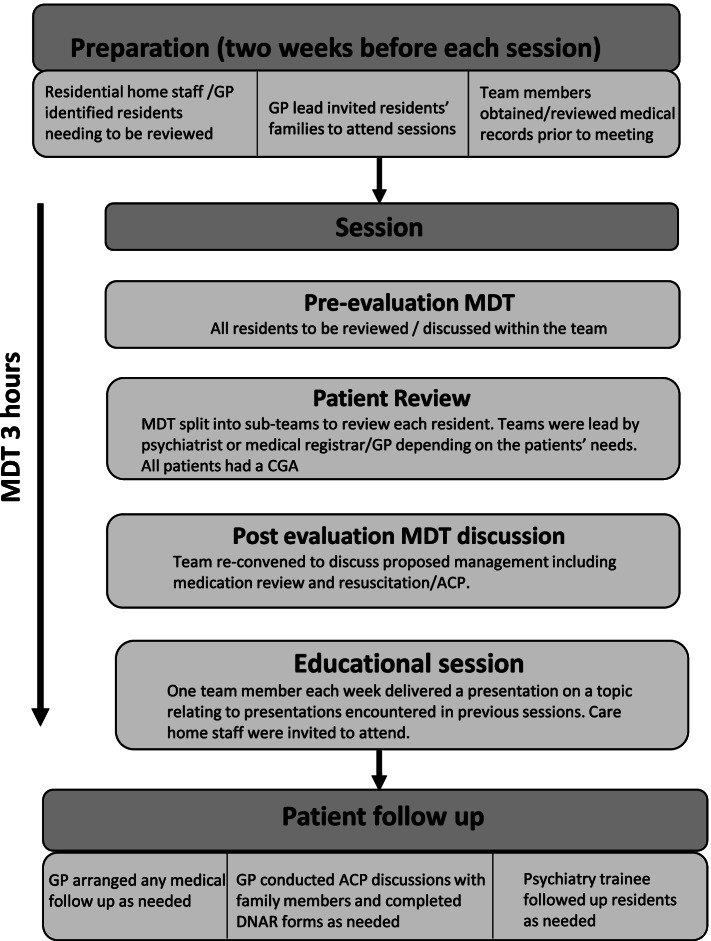
Design for each monthly session

MDT reviews were held in the RH (Fig. 1). Residents whose physical or mental health caused concern raised by care home staff or following GP review were identified in advance and families were invited to attend. Team members obtained medical records in advance. Sessions lasted 3 h. Pre-evaluation MDT discussion was followed by clinical assessment. The team divided into smaller groups to review residents according to whether there was a geriatric, psychiatric or general medical focus. The team re-convened to discuss proposed management, including medication review and identified an educational topic for the following session based on predominant issues. Following sessions, GPs made any treatment changes, organised follow-up and held resuscitation and ACP discussions with families. Psychiatric follow-up occurred where necessary.

### Cycle 2

In cycle 2, a GP trainee took over the lead role to encourage trainee leadership. The team expanded to include more members ensuring there was at least one member from each discipline in each session.

### Outcomes

To assess intervention impact, we collected data relating to the project aims as defined by the EHCH framework; CGA, polypharmacy, end-of-life care and education. Outcome measures were reviewed following cycle 1 and some were adapted for cycle 2. To ensure data collection was complete, we had full access to all GP and RH records. We monitored the local health and social care economy for any other initiatives or changes in service provision which may have impacted our project.

### Individual resident level data

We collected data on demographics including age, gender and clinical factors including number and type of medications [17] (but not classification at the time of being assessed), physical and psychiatric comorbidities as documented for each patient on the NHS summary care record (this is a list of comorbidities held for each patient in UK primary care) and Clinical Frailty Scale (CFS). The CFS has established reliability and validity [18] and helps identify patients with severe frailty to trigger appropriate interventions such as advance care planning. The CFS ranges from 1-being very fit to 9-being terminally ill. We documented whether participants had capacity to decide about the treatment plan.

### System level data

We collected data from GP records on the number of hospital admissions from the RH (in cycle 1 admission data were collected during the project only and in cycle 2 admission data were collected in the 12 months prior, during and 12 months after the project). Costs of acute admissions for the whole RH were obtained for cycle 2 from the CCG, calculated using Healthcare Resource Groups. These are standard groupings of clinically similar treatments which use common levels of healthcare resource. This is a validated method to determine fair and equitable reimbursement for healthcare services in the UK [19].

### Process measures

In cycle 1, feedback from participating teams was gathered independently by an education group formed by Health Education England (HEE) and University College London Partners (UCLP). Educational sessions were assessed by documenting the topics, number of attendees, RH staff feedback via questionnaire (cycle 2 only), and participant feedback (in cycle 1 feedback was from HEE and UCLP and in cycle 2 feedback was via questionnaire). We monitored return rates of questionnaires.

### Data analysis

This was a quality improvement project we therefore used simple descriptive statistics for data collected. For example, mean age of participants, number of medications, number of comorbidities were described with mean and range. Simple percentages were used to described outcome data such as advance care plans and number of DNACPR forms. We calculated the mean session rating based on MDT feedback. We also extracted qualitative data using comments from the MDT feedback forms.

### Ethical considerations

This was a quality improvement project, sponsored by HEE and UCLP who are part of the Academic Health Science Networks; driving adoption and spread of innovation across healthcare. The project was led by local GPs and was an enhancement of their usual service. The implementation of the project was monitored by HEE. Staff were given the option of whether they wished to complete questionnaires or not.

## Results

The RHs ranged between 21 and 73 beds (Table 1). Tables 1 and 2 show resident demographics and outcomes.

**Table 1:** Care home and resident demographics

	Cycle 1		Cycle 2
	Care Home 1	Care Home 2	Care Home 3
Location	North London (urban)	North London (urban)	North London (urban)
Type of care	Residential	Residential	Residential
Beds	21	35	73
Care Quality Commission (CQC) rating (date of inspection)	‘Good’ (July 2017)	‘Good’ (October 2017)	‘Inadequate’ (December 2018)
Number of sessions held	5	2	10
Dates of sessions	Sept 2017 – Jan 2018	Nov 2017 – Feb 2018	March 2019 – Dec 2019
Number of residents assessed	9	4	21
Mean age (range)	82.1 (62–95)	80.8 (78–84)	86.4 (77–97)
Male: female	5:4	0:4	11:10
Mean number of medications (range)	9.0 (4–18)	10.0 (5–12)	7.1 (2–18)
Mean number of comorbidities excluding dementia (range)	7.8 (4–13)	9.3 (6–15)	6.7 (3–16)
Number with pre-recorded dementia diagnosis (% of total participating residents)	7 (77.8%)	4 (100%)	16 (76.2%)
Number of new diagnoses of dementia made during intervention (% of total participating residents)	0 (0%)	0 (0%)	0
Number of residents with BPSD (% of participating residents with dementia)	3 (42.9%)	4 (100%)	16 (100%)
Mean clinical frailty score (range)	6.6 (6–8)	6.0 (5–7)	7.3 (6–9)
Number with capacity to decide about the treatment plan (% of total participating residents)	1 (11.1%)	0 (0%)	4 (19.0%)

**Table 2:** Data outcomes

	Cycle 1		Cycle 2
	Care Home 1	Care Home 2	Care Home 3
Presenting complaints	Stealing other residents’ food in context of dementia Worsened cough and breathlessness Cognitive decline and refusal of medications including warfarin Aggression and paranoia Breathlessness and ankle swelling Weight loss, day time drowsiness Uncommunicative, decline in mobility, decline in oral intake Pain and poor mobility Falls	Sleeping in chair, leg pain and swelling Anxiety, agitation Recurrent UTI, generally unwell and has been in bed for a no. of weeks Rash, hallucinations and delusions	Weight loss Difficulty breathing Challenging behaviour Swollen legs Refusing medication General decline possible depression Reduced oral intake Reduced mobility Low mood, insomnia Slowing and movement disorder Falls Depression in context of dementia New patient needing CGA Loose stool, anaemia
Number of GCA reviews held	9	4	21
Mean change in number of medications (range)	−1.4 (−3 to + 1)	−0.3 (− 1 to 0)	−2.1 (− 18 to + 1)
Change in BPSD (staff report)	3/3 improved	1/4 improved 3/4 no different	Data not collected in this cycle
Change in BPSD (family report)	3/3 improved	2/4 improved 2/4 no different	Data not collected in this cycle
Number of reviews attended by relatives (% of total participating residents)	1 (11.1%)	0 (0%)	1 (4.8%)
Number of residents with ACP initiated or reviewed (% of total participating residents)	7 (77.8%)	3 (75%)	16 (76.2%)
Number of DNAR forms completed or reviewed pre- and post- MDT (% of total participating residents)	6 newly initiated (66.7%)	2 newly initiated (50.0%)	Pre-MDT = 4 (19%) Post-MDT = 19 (90.5%)
Number of patients with Coordinate My Care record created (% of total participating residents)	0 (0%)	0 (0%)	8 (38.1%)
Project period (months)	7	3	10
Actual number of admissions to hospital during the project period in intervention residents (no. per month)	1 (0.16)	0 (0.0)	0 (0.0)
Number of admissions among participating residents in 12 months prior to reviews (mean per person over 12 months)	Data not collected in this cycle	Data not collected in this cycle	25 (1.19)
Number of admissions among participating residents in 12 months post review (mean per person over 12 months)	Data not collected in this cycle	Data not collected in this cycle	6 (0.29)
Reduction of admissions among participating residents over 1 year (mean per person over 12 months)	Data not collected in this cycle	Data not collected in this cycle	19 (0.9)
Costs of hospital admissions for the whole care home	Data not collected in this cycle	Data not collected in this cycle	12 months prior to intervention £55,678 During year of intervention £49,653
Number of educational sessions	5	2	9
Topics of half hour educational sessions	Polypharmacy and medication errors Challenging behaviour in dementia Nutrition Pain in dementia Difficult ACP discussions	Dementia pre-diagnosis counselling Cardiovascular complications in geriatrics	Skin care in the older patient Advance care planning Nutrition and weight loss Morbidity and mortality meeting End of life in the care home Old age psychiatry Behavioural symptom management Rationalisation of medications Communication between primary and secondary care

### Cycle 1

#### Process

A total of 13 residents were assessed across seven sessions; 5 males and 8 females, mean 82.5 years old; mean number comorbidities 8.2; mean number medications 8.6, mean clinical frailty score 6.5 (moderately frail) (Table 1). Only 1(11%) resident had mental capacity (determined by the 2005 Mental Capacity Act) [20] to engage in discussion about their treatment plans. For the remainder, plans were made in best interests. No residents declined to participate or dropped out of the project, i.e. through change of residence.

#### CGA

All 13 residents had a CGA with psychiatric input and 11 (85%) had confirmed diagnosis of dementia; 7 (54%) with behavioural and psychological symptoms of dementia (BPSD) or a secondary psychiatric problem. Of those with BPSD, 4 (57%) (according to staff) or 5 (71%) (according to families) improved from the point of intervention over the subsequent 6 months. Three residents had acute medical issues addressed, potentially avoiding hospital admissions; urinary tract infection, chronic obstructive pulmonary disease exacerbation and fluid overload. One resident required urgent admission. No other residents were admitted during the project’s course.

#### Polypharmacy

Reviews resulted in an overall reduction of − 0.8 medications per resident. This was achieved using STOPP START criteria [21] and checking anticholinergic burden.

#### End of life care plans

Families were unable to attend for all but one resident. A total of 10 separate ACP discussions were held by GPs with families, triggered by the MDT. All residents were suitable for Do Not Attempt Cardio-Pulmonary Resuscitation (DNACPR) and ACP; 8 were newly initiated. ACPs are now coded as a ‘significant problem’ in the GP summary care record ensuring it is easily visible in the main section of the summary.

#### Education

A total of 7 half-hour educational sessions were delivered (Table 2). We took a participatory approach and the topics were decided collectively by the MDT based on interesting cases the previous week and areas of education felt to be needed by the team.

### Learning and adaptation from cycle 1

After cycle 1 the multidisciplinary group met to review all 13 cases focusing on the interventions made, the outcomes, the end-of-life care plans and the feedback from the educational sessions. We reflected on the barriers and facilitators to our achieving our objectives. From cycle 1 we understood the value of making decisions as a team and inter-professional learning, so continued this model in cycle 2. To promote the training aspect of the project, the GP trainee was the team lead in cycle 2. Trying to cover two RHs was difficult and reduced continuity. We therefore focused on one home for cycle 2. In cycle 1 relatives were mostly unable to come to meetings so we did not set this as a priority, rather we highlighted those needing further discussion. The educational value of the sessions was more apparent after cycle 1 and so medical, pharmacy and other students were invited to attend. Unlike in cycle 1, data on BPSD was not collected in cycle 2 as it was not central to our aims but we did include hospital admission data pre and post project. We also gathered data on electronic ‘Coordinate my Care’ (CMC) records created in cycle 2 as a result of learning in cycle 1. CMC is a London-wide online platform to share advance care planning information between healthcare providers.

### Cycle 2

#### Process

A total of 21 residents were assessed in ten sessions, (11 males and 10 females, mean age 86.4 years old; mean number comorbidities 6.7; mean number of medications 7.1, mean clinical frailty score 7.3). Four (19%) patients who had capacity for medical decisions. No residents declined to participate or dropped out of the project, i.e. through moving out of the RH.

#### CGA

All 21 residents had CGA with psychiatric input and 16 (76.2%) had dementia with evidence of BPSD at review. For the residents reviewed there was a mean reduction in admissions of 0.9 per person per year (see Table 2). Acute hospital admission costs for the whole home were reduced by £6025 during the year of intervention compared to the previous year.

#### Polypharmacy

All 21 residents had a medication review with a mean reduction of − 2.1 medications per resident.

#### End-of-life care plans

All 21 residents had resuscitation status reviewed and ACPs were initiated or reviewed for 16 (76.2%) residents with the input from family where appropriate. An independent mental capacity advocate was needed for one resident. There were eight (38.1%) residents with a new care plan created on CMC, previously no residents had a CMC record.

#### MDT feedback

The members of the MDT all strongly agreed that they had benefited from collaborative working and learning as part of an MDT with 100% agreeing their confidence had grown in managing residents with psychological and behavioural problems, deprescribing medications and end of life discussions. All members of the MDT agreed the project had improved their understanding of primary and secondary care systems. Feedback comments included “*useful to learn from other specialties”, “most enjoyed understanding roles and responsibilities of everyone involved in the care of a resident,” “I enjoyed bridging the gap between primary, secondary and psychiatric care”.* Staff comments from feedback questionnaires commented *feeling “much more supported in the care home with the presence of the MDT,”* and finding it *“easier to raise any problems the residents may be having as the team are more accessible.”*

## Discussion

Our objective was to improve multidisciplinary care in RHs, specifically focussing on CGA, medication burden, end-of-life care planning and team education. Our detailed MDT assessment of 34 residents from three RHs reduced prescribed medications and acute hospital admissions and thus NHS costs. We increased the completion of DNACPR forms and our educational sessions received positive feedback.

CGA improves outcomes for older people in the community, hospital setting and long-term facilities [22–24] but is not routinely conducted in RHs [25].

We implemented our model specifically in RHs to deliver CGA with input from GP, geriatricians, psychiatrist, pharmacist, RH staff/ managers and family members when available. We did not have direct access to therapists, social workers and voluntary staff but the GP liaised with these services when needed. Psychiatric input was essential; over 75% of residents had dementia and a high proportion experienced BPSD. Other initiatives have also found psychiatric support vital. In Camden and Islington the MDT has actively focused on mental wellbeing with psychology resources and activity coordinators, increasing knowledge, skills and staff support resulting in fewer hospital admissions and shorter lengths of stay [26]. Our project reflects similar ongoing work in the UK such as the Proactive Healthcare of Older People in Care Homes (PEACH) protocol which uses a quality improvement collaborative (QIC) intervention to improve the delivery of CGA in care homes. The PEACH intervention team comprises a GP, social care staff, nursing staff, therapists, geriatricians, voluntary staff, pharmacists, dementia specialists, care home workers/mangers and members of the public [27]. Relational working between the care home and external services is key to successful healthcare delivery in this setting [28].

Our MDT approach resulted in fewer emergency admissions and reduced medication burden. Staff reported feeling more supported and were more proactive in bringing issues to our attention. The educational sessions were open to all staff. Our intervention may have had wider impact across the RH as there was a reduction in hospital admission costs across the whole home.

There is little literature on addressing polypharmacy in RHs where residents are particularly vulnerable to inappropriate prescribing [29]. One systematic review showed that MDT meetings, educational interventions, particularly face-to-face education improved prescribing quality [30]. Our intervention facilitated MDT discussions and educational sessions around polypharmacy. Specialist pharmaceutical input also helped to reduce medications prescribed, potentially reducing costs.

Care homes (including RHs) will become the commonest place of death over the next 20 years [31]. Education, particularly peer-training and inter-professional collaboration are potentially effective mechanisms for improving end-of-life care, although education for care home staff with a high turn-over would need to be ongoing to have a sustainable impact [32]. We dedicated 3 of 16 education sessions to end-of-life care. We had multiple discussions as an MDT around end-of-life for residents enabling the development of patient-centred care plans and improved group knowledge and experience [33]. As a result of the project one GP practice to set up an ‘ACP clinic’. Within both cycles, most residents had a DNACPR form completed. In cycle 2, we used an online electronic system (Coordinate My Care) to share care plans with GPs, secondary care and the Ambulance Service.

There are limitations to the model developed. Ideally MDT participants should not change however this is inevitable when using specialist trainees who regularly rotate. The presence of the same GP lead, pharmacist and RH staff facilitated continuity. It is important when delivering CGA as part of an MDT that there is strategic collaboration between organisations providing team members, to ensure effective MDT functioning [22]. For sustainability, trainees require protected time away from regular duties. With multiple RHs, more trainees would be required, drawing resources away from secondary care. Standardised proformas to facilitate CGA would have reduced variability and improved outcome monitoring. In addition, the MDT did not include therapists or social workers who could add considerable value. Systematic processes for screening residents in need of review such as medication burden or hospital admissions, may be more effective. Reducing acute admissions shifts the burden of care onto the RH, i.e. people who die there may have previously gone to hospital, nursing home or hospice creating increased emotional burden on staff.

We only saw a proportion of residents and would need more sessions to review all. Few family members could attend, which could have led to under-representation of patients’ values and preferences. With more organisation, families could be invited earlier saving GPs time in following up with them. Data gathered regarding patient reviews and staff feedback differed between cycles as the project developed, making it harder to compare outcomes. We took a pragmatic approach to data collection but there are some limitations to this for example we only counted medications but did not look at groups and types of medications. This quality improvement project was set across three care homes so may not be generalisable to other settings.

Whilst this project was running there were widespread changes in policy, for example the primary care networks (PCN) UK national rollout in 2020 which ensured that every care home is now supported by an MDT by their PCN. Our project aligns with this change in policy as we have shown the benefits of the MDT working within the care home. As well as policy changes there are other emerging studies in this field such as the GRAPE study which will be looking at how GPs are involved in initiatives to improve services and care within the care home setting [34].

The EHCH framework highlights variable access for care home residents to NHS services [16] but does not specifically mention mental health. Our project supports the Royal College of Psychiatrists report on delivering the Long-Term Plan advocating mental health input as central to care home services [35]. The BGS policy calls for access to CGA, personalised care plans and follow-up for all older people with frailty, dementia, complex and long-term conditions. Our intervention provides a mechanism to deliver on these policies, creating an opportunity for shared learning and enabling residents to receive more specialist care.

## Conclusion

This was an effective multi-disciplinary project which facilitated CGA within the RH setting whilst focussing on reducing polypharmacy and improving end-of-life care. This had a perceived impact on both physical and mental health of residents. The MDT were able to benefit from the shared learning opportunities and improved inter-professional relationships. This project demonstrates a sustainable model which could be applied to other RHs.

## Data Availability

The datasets used and/or analysed during the current study available from the corresponding author on reasonable request.

## Abbreviations

RHs: Residential Homes
ACP: Advance care planning
EHCH: Enhanced Health in Care Homes
NHS: National Health Service
MDT: Multi-disciplinary team
BGS: British Geriatrics Society
CGA: Comprehensive Geriatric Assessment
GP: General Practitioner
CQC: Care Quality Commission
HEE: Health Education England
UCLP: University College London Partners
BPSD: Behavioural and psychological symptoms of dementia
DNACPR: Do Not Attempt Cardio-Pulmonary Resuscitation.
CMC: Coordinate My Care
PEACH: Proactive Healthcare of Older People in Care Homes
QIC: Quality improvement collaborative
PCN: Primary Care Network
